# Epigenetic Modulation of Microglial Inflammatory Gene Loci in Helminth-Induced Immune Suppression: Implications for Immune Regulation in Neurocysticercosis

**DOI:** 10.1177/1759091415592126

**Published:** 2015-07-02

**Authors:** Arun Chauhan, Fredice Z. Quenum, Ata Abbas, David S. Bradley, Sergei Nechaev, Brij B. Singh, Jyotika Sharma, Bibhuti B. Mishra

**Affiliations:** 1 Department of Basic Sciences, School of Medicine and Health Sciences, The University of North Dakota, Grand Forks, ND, USA

**Keywords:** helminth, microglia, immune suppression, epigenetics, neurocysticercosis

## Abstract

In neurocysticercosis, parasite-induced immune suppressive effects are thought to play an important role in enabling site-specific inhibition of inflammatory responses to infections. It is axiomatic that microglia-mediated (M1 proinflammatory) response causes central nervous system inflammation; however, the mechanisms by which helminth parasites modulate microglia activation remain poorly understood. Here, we show that microglia display a diminished expression of M1-inflammatory mediators such as tumor necrosis factor-alpha (TNF-α), interleukin-6 (IL-6), and nitric oxide synthase 2 (NOS2) in murine neurocysticercosis. Microglia also exhibited a lack of myeloid cell maturation marker major histocompatibility complex (MHC)-II in these parasite-infected brains. Treatment of microglia with helminth soluble/secreted factors (HSFs) *in vitro* did not induce expression of M1-inflammatory signature molecule NOS2 as well as MHC-II in primary microglia. However, HSF treatment completely inhibited lipopolysaccharide-induced increase in expression of MHC-II, NOS2 and nitric oxide production in these cells. As epigenetic modulation of chromatin states that regulates recruitment of RNA polymerase II (Pol-II) is a key regulatory step in determining gene expression and functional outcome, we next evaluated whether HSF induced modulation of these phenomenon in microglia *in vitro*. Indeed, HSF downregulated Pol-II recruitment to the promoter region of TNF-α, IL-6, NOS2, MHC-II, and transcription factor CIITA (a regulator of MHC-II expression), by itself. Moreover, HSF suppressed the lipopolysaccharide-induced increase in Pol-II recruitment as well. In addition, HSF exposure reduced the positive histone marks H3K4Me3 and H3K9/14Ac at the promoter of TNF-α, IL-6, NOS2, MHC-II, and CIITA. These studies provide a novel mechanistic insight into helminth-mediated immune suppression in microglia via modulation of epigenetic processes.

## Introduction

Infection of the central nervous system (CNS) by the cestode *Taenia solium* (*T. solium*) results in neurocysticercosis (NCC), the most common parasitic disease affecting 50 to 100 million people worldwide (White, 2000). Signs and symptoms of NCC may include the following: seizures, strokes, hydrocephalus, and symptoms associated with increased intracranial (i.c.) pressure (Nash et al., 2006). Indeed, NCC is the number one cause of epilepsy and contributes to as many as half of the total adult-onset seizures (White, 1997; Garcia and Del Brutto, 2005). Effective treatment for NCC remains a challenge, as the severity of disease symptoms is thought to be a result of pathologic inflammatory response induced by the degenerating larvae (Roman et al., 2000; White, 2000; Sciutto et al., 2007). Interestingly, NCC is typically associated with a long initial asymptomatic phase lasting several years before the onset of symptomatic phase (White et al., 1997; White, 2000; Restrepo et al., 2001). This asymptomatic phase is correlated with a lack of detectable inflammation in the CNS, presumably due to the ability of the cysticerci to induce immune suppression (White et al., 1997; White, 2000; Restrepo et al., 2001). Indeed, in NCC patient brains, a lack of surface expression of activation/maturation marker major histocompatibility complex (MHC)-II in macrophages has been documented in areas around the larvae (Alvarez et al., 2008). Thus, parasite-derived molecules likely play an important role in suppression of inflammation in the CNS helminth infection. Understanding these immune suppressive mechanisms may help develop new ways of therapeutically regulating CNS inflammation in NCC and other pathological condition.

Inflammation in the CNS in response to diverse stimulus, for example, infection, injury, or trauma, is mainly regulated by the innate immune response (Ramesh et al., 2013a; Kielian, 2014). Among the nervous tissue cell types, microglia serve as the primary innate immune cell providing first line of response (Rivest, 2009; Cherry et al., 2014). When activated, microglia express a number of genes and development of inflammatory responses, depending on the nature, intensity, and duration of the insult. The functional phenotypes of microglia vary in response to external stimuli these cells receive through a wide variety of surface receptors. Microglia displaying the classical activation phenotype (M1) release multiple proinflammatory cytokines and generally promote the destruction of pathogens, but also cause widespread bystander tissue damage as a result of their overactivation (Kobayashi et al., 2013). However, the mechanisms underlying helminth-induced suppression of M1-inflammatory signature in microglia are not clearly understood.

Microbial products (e.g., lipopolysaccharide [LPS]) or endogenous inflammatory stimuli (e.g., interferon gamma) activate myeloid innate immune cells types, such as microglia and macrophages, to produce M1-inflammatory mediators (e.g., antimicrobial peptides, reactive nitrogen and oxygen species, chemokines and cytokines; Mosser and Edwards, 2008; Gordon and Martinez, 2010; Lawrence and Natoli, 2011; Murray and Wynn, 2011; Kobayashi et al., 2013). Sensing of such stimuli by surface receptors leads to activation of downstream NF-κB (Nuclear Factor Kappa B), MAPK (Mitogen-activated protein kinase), and JAK-STAT (Janus kinase/signal transducers and activators of transcription) signaling pathways (Takeuchi and Akira, 2010) that have been found to integrate into a coherent pattern of changes in *epigenetic landscape* and subsequent gene transcription (Natoli et al., 2011). Indeed, genome-wide analyses have clearly shown that epigenetic chromatin marks are dynamically regulated in response to diverse stimuli (Zhou et al., 2011). The expression of genes are associated with functionally distinct polarizing class of histone marks (Ernst et al., 2011), for example, positive histone marks, H3K4me3 and H3K9/14Ac are associated with actively transcribed genes, whereas H3K27me3 are associated with gene repression (Medzhitov and Horng, 2009; Smale, 2010). In stimulated cells, these events involving chromatin marks regulate accessibility around the promoter region and facilitate RNA polymerase (Pol) II recruitment to the promoter (Adelman et al., 2009). The cascade of events that follows ultimately leads to Pol-II elongation and productive RNA synthesis (Adelman et al., 2009). *In toto*, epigenetic events involving chromatin marks contribute to Pol-II recruitment to the gene promoter, and Pol-II-dependent transcription elongation is important for gene transcription (Rogatsky and Adelman, 2014). An important question, therefore, is whether helminths induce modulation of epigenetic mechanisms involving chromatin marks, Pol-II recruitment, and elongation that are involved in the inhibition of inflammatory responses in microglia. This is an understudied area of research.

In the present study, using a murine model for NCC developed by i.c. infection of mice with *Mesocestoides corti* (*M. corti*) metacestodes (Mishra et al., 2008, 2009b; Alvarez et al., 2010a; Mishra et al., 2010; Gundra et al., 2011), the maturation and activation phenotype of microglia was determined by the expression of MHC-II, CIITA, tumor necrosis factor-alpha (TNF-α), interleukin-6 (IL-6), and nitric oxide synthase 2 (NOS2) in parasite-infected brains. To determine whether epigenetic events of microglia activation and maturation are affected by the parasite, we tested the effect of helminth soluble/secreted factors (HSFs) on expression of positive histone marks H3K4me3 and H3K9/14Ac, and Pol-II recruitment to promoter of immune molecules by Chromatin Immunoprecipitation (ChIP) analysis. Moreover, effect of HSF (Sun et al., 2014) on toll-like receptor (TLR)-induced upregulation of H3K4me3 and H3K9/14Ac expression, and increased Pol-II recruitment at the promoter of these immune mediators was analyzed. Our results presented here show that the regulation of epigenetic pathways by helminth molecules is likely a novel immune suppressive mechanism to block microglia-associated M1-inflammatory response in NCC.

## Methods

### Animals and Antigens

Maintenance of the animals and tissue collection for all studies presented here were conducted under the guidelines of the University of North Dakota’s IACUC, the U.S. Department of Agriculture, and the National Institutes of Health. Female BALB/c and C57BL/6 mice used in this study were bred and maintained in the animal facility at the University of North Dakota SMHS, Grand Forks, ND. *M. corti* metacestodes were propagated in the peritoneal cavity of BALB/c mice by serial intraperitoneal (i.p.) infection. HSF consisting of *M. corti* soluble/secreted factors was prepared from *M. corti* metacestodes by freezing and thawing as described previously (Chauhan et al., 2014; Sun et al., 2014).

### Antibodies

The following antibodies were used to analyze microglia activation and maturation: M1/70 (anti-Mac1), 1D3 (anti-MHC-II), as well as purified anti-mouse TNF-α, IL-6, and NOS2 (BD Biosciences, San Diego, CA). For immunofluorescence (IF) staining, fluorescent-conjugated secondary antibodies and isotype control antibodies (Jackson Immuno Research Laboratories, West Grove, PA) were used. Antibodies used in ChIP assays included anti-Pol-II (Santa Cruz Biotechnology [Dallas, Texas, USA]) at 2 mg/IP, anti-H3K4Me3 (Millipore [Billerica, Massachusetts, USA]) at 1 mg/IP, anti-H3K9/14Ac (cell signaling technology [Danvers, Massachusetts, USA]) at 2 mg/IP, or normal rabbit IgGs.

### Histology and IF Staining in Brains During Murine NCC

Murine NCC was induced by i.c. injection of 50 μl of HBSS (Hank's balance salt solution) containing approximately ∼40 *M. corti* metacestodes into 5-week old C57BL/6 mice under short-term anesthesia (Cardona et al., 1999; Cardona and Teale, 2002; Mishra et al., 2006, 2009; Chauhan et al., 2014). Mock-infected control mice were sham injected with 50 μl sterile HBSS alone. At indicated times postinoculation, anesthetized animals were perfused through the left ventricle with 20 ml cold phosphate-buffered saline. The brains were immediately removed from perfused animals, embedded in O.C.T. resin, and snap frozen. Serial horizontal cryosections, 10 µm in thickness, were placed on silane prep slides (Sigma-Aldrich, St. Louis, MO). The slides were air-dried overnight and fixed in fresh acetone for 20 s at room temperature. Acetone-fixed sections were wrapped in aluminum foil and stored at −80℃ or processed immediately for *in situ* IF microscopy analysis as previously described (Cardona et al., 2003; Mishra et al., 2006; Alvarez and Teale, 2007a, 2007b). To rule out any nonspecific staining, several control experiments were performed. In each case, sections were blocked with saturating concentrations of appropriate isotype control antibodies or host serum antibodies to eliminate FcR-mediated nonspecific binding. In addition, staining in the absence of primary antibodies was performed as negative controls.

### Primary Microglia Maturation and Activation

Microglia were purified from postnatal Day 1 (P1) mouse brains (C57BL/6) as previously described (Floden et al., 2005; Nagamoto-Combs and Combs, 2010; Sun et al., 2014). Briefly, meninges-free cortices from P1 mice were isolated and trypsinized. Cells were plated onto tissue culture plastic in Dulbecco’s modified Eagle’s medium (DMEM)-F-12 with L-glutamine (Invitrogen [Waltham, Massachusetts, USA]) containing 10% heat-inactivated fetal bovine serum and 5% heat-inactivated horse serum and fed every third day. After ∼14 days, the cultures were shaken vigorously (30 min; 120 rpm on a rotary shaker) to remove microglia. Microglia purity was routinely determined to be ∼90% cells by IF microscopy using specific markers IBA-1 or CD11b and negative staining for glial fibrillary acidic protein (a marker for astrocytes). The cells were plated at 1.5 × 10^6^ per well in six-well plates and were incubated for 24 hr at 37℃ in medium alone, or in the presence of HSF, LPS, or HSF/LPS. After 24 hr poststimulation, microglia were surface stained with anti-MHC-II to measure expression of this maturation marker using a BD LSR II flow cytometer (BD Biosciences) as we have previously described (Chauhan et al., 2014). The gating scheme included discrimination of single cells from doublets by plotting linear forward-scatter height versus forward-scatter area. Live microglia cells were then selected by plotting side scatter versus the Am Cyan channel, which included the live/dead stain (live gate). From the live gate, MHC-II + cells were selected via side scatter versus Alexa 700 channel. Gates for MHC-II + cells were set based on isotype control staining. FlowJo v7.6 (Treestar) was used for all flow cytometry analyses.

To test the inhibitory effect of HSF on production of nitric oxide (NO), the cells were plated at 8 × 10^4^ cells per well in 96-well flat-bottom plates and were stimulated with medium alone, or in the presence of HSF, TLR ligand LPS, or HSF/LPS. Culture supernatants were collected 24 hr after stimulation, and NO production was measured by using the Griess reagent (Promega [Fitchburg, Wisconsin, United States]) according to the manufacturer’s instructions.

### ChIP Experiments

ChIP experiments were carried out as previously described (Dhasarathy et al., 2007; Adelman et al., 2009). Briefly, microglia were seeded into 15-cm dishes and were pulsed with medium alone, or in the presence of HSF, LPS, HSF/LPS for 24 hr at 37℃. Cells were cross-linked with formaldehyde for 10 min at room temperature, followed by addition of 125 mM glycine for 5 min at 4℃ for quenching formaldehyde. Cells were washed in ice-cold phosphate-buffered saline and harvested. Cells were lysed by sonication with a previously determined optimal cycle conditions to generate 250- to 150-bp DNA fragments using Covaris Sonicater. Immunoprecipitation was performed with the antibodies indicated, and IgG was used as a control. Precipitated DNAs were detected by polymerase chain reaction (PCR) using specific primers. Sequences of the specific primers used for IL-6, NOS2, TNF-α, H2Eβ (MHC-II domain in C57BL/6), CIITA, and GAPDH are as follows: IL-6 (sense) 5′-ACTCCTCTCTCACAGTCTCAATA-3′ and (antisense) 5′-GGGATGTCTGTAGCTCATTCTG-3′; NOS2 (sense) 5′-TCCCTAGTGAGTCCCAGTTTTGA-3′ and (antisense) 5′-CTGGTCGCCCGTCCAAGG-3′; TNF-α (sense) 5′-CCCCAACTTTCCAAACCCTCT-3′ and (antisense) 5′-CCCTCGGAAAACTTCCTTGGT-3′; H2Eβ (MHC-II) (sense) 5′-AAACAACCCAAAGCAAAACC-3′ and (antisense) 5′-TCAGCATCAAAGGAGTCCAG-3′; CIITA (sense) 5′-TGCCTTTGGCCCAAAGCTGAA-3′ and (antisense) 5′-TTCTGAGTGCTGCCTGCATGC-3′; and GAPDH (Active Motif Cat # 71018). Real-time PCR analysis on respective immunoprecipitated DNA was performed using SYBR green as the detection dye (Mishra et al., 2008). The percent input for each sample was calculated based on a standard curve using 10%, 1%, 0.1%, and 0.01% of input DNA. Precipitated DNA with anti-IgG served as a background control.

### Statistical Analysis

Student’s *t* test and one-way analysis of variance was used to compare the means of different groups (SIGMA PLOT 8.0 [Systat Software, San Jose, CA]). A *p* value of less than .05 was considered to be statistically significant.

## Results

### Microglia in Murine NCC Brains Exhibit Attenuated Inflammatory Phenotype/Profile

**Figure 1.**
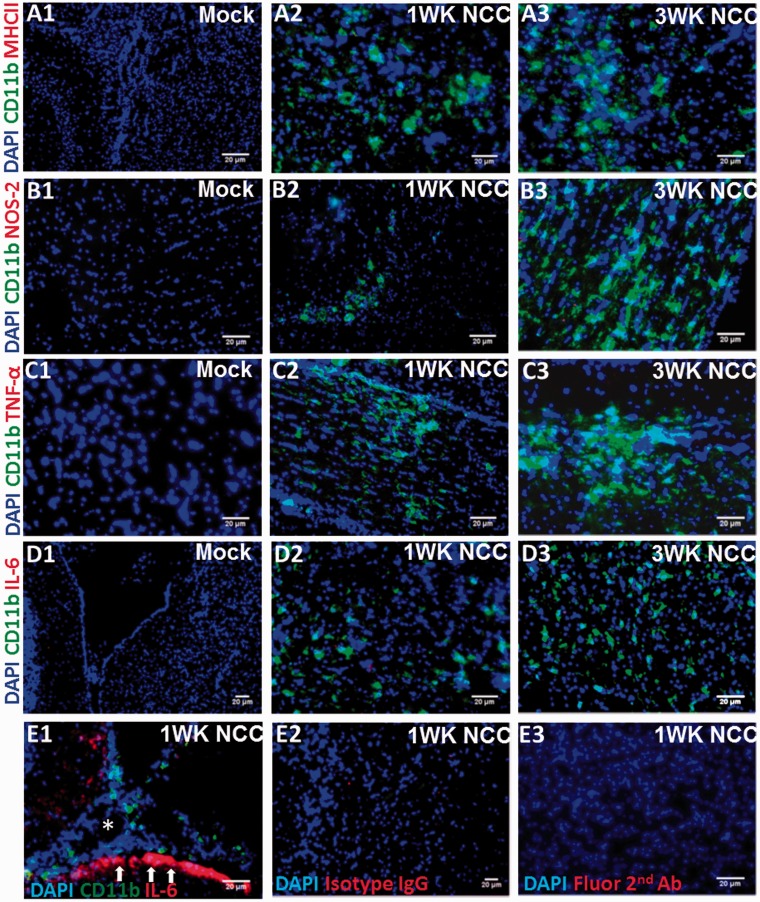
Microglia display reduced expression of inflammatory mediators in murine NCC. C57BL/6 mice were i.c. infected with *M. corti* or HBSS (mock) and sacrificed at various times p.i. Frozen brain cryosections at 1 week p.i. were analyzed by *in situ* IF staining for expression of inflammatory mediators MHC-II, TNF-α, IL-6, and NOS2 using specific fluorochrome-conjugated antibodies. CD11b + microglia in brain parenchyma are depicted in green. Nuclear staining DAPI is depicted in blue. Mock-infected control brains double IF stained with anti-CD11b and immune mediators MHC-II (A1) (cortex cerebri parietalis; 200×), NOS2 (B1) (cortex cerebri; 200×), TNF-α (C1) (cortex cerebri; 200×), and IL-6 (D1) (stratum griseum; 100×). *M. corti* infection at 1 week showing CD11b + microglia with an undetected/diminished stating of MHC-II (A2) (cortex cerebri occipitalis; 200×), NOS2 (B2) (cortex cerebri; 200×), TNF-α (C2) (cortex cerebri; 200×), and IL-6 (D2) (stratum griseum; 200×). *M. corti* infection at 3 week showing CD11b + microglia with an undetected/diminished staining of MHC-II (A3) (cortex cerebri parietalis; 200×), NOS2 (B3) (cortex cerebri; 200×), TNF-α (C3) (cortex cerebri; 200×), and IL-6 (D3) (stratum griseum; 200×). (E1) *M. corti* infection at 1 week showing IL-6 positive staining in brain anatomical region in proximity to the meninges (200×). The arrow points to the positive staining in cells morphologically similar as astrocytes and its processes. (E2) Isotype control IgG showing a lack of staining in *M. corti*-infected brain at 1 week p.i. (stratum griseum; 100×). (E3) Negative control staining with fluorescent-conjugated secondary Ab in the absence of the primary antibody in *M. corti*-infected brain at 1 week p.i. (stratum griseum; 100×). NCC = neurocysticercosis; i.c. = intracranial; IF = immunofluorescence; MHC = major histocompatibility complex; TNF-α = tumor necrosis factor-alpha; IL-6 = interleukin-6; NOS2 = nitric oxide synthase 2.

The kinetics of microglial expression of MHC-II, TNF-α, IL-6, and NOS2 were compared by *in situ* IF microscopy in brain tissues from mock-infected and NCC mice. Microglia were identified by IF microscopy using cell markers CD11b together with their characteristic morphology and location in brain parenchyma. In normal physiological conditions, microglia exhibit resting/quiescent phenotypes with low basal level expression of CD11b, while an increased CD11b expression is among the earliest signs of microglial response (Frick et al., 2013). As expected, the CD11b expression was detected at a low/undetected basal level in microglia in mock-infected brains as compared with the parasite-infected brains (Figure 1). Importantly, the expression of the M1-inflammatory molecules, MHC-II, NOS2, TNF-α, and IL-6 were minimally detected in the brain of mock-infected animals (Figure 1(A1), (B1), (C1), and (D1)). In the parasite-infected brains, expression of MHC-II (Figure 1(A2)), NOS2 (Figure 1(B2)), TNF-α (Figure 1(C2)), or IL-6 (Figure 1(D2)) proteins were barely detected or undetected in microglia cells in brain parenchyma at 1 week, and 3 week p.i. (Figure 1(A3), (B3), (C3), and (D3)). However, IL-6 was upregulated in cells with morphology similar to those of astrocytes localized to the brain anatomical region in proximity to the meninges (Figure 1(E1)), suggesting a microglia-specific immune suppression. IF staining using isotype control Ab or staining in the absence of primary antibodies (only fluorescent-conjugated secondary Ab) did not show any signal (Figure 1(E2) and (E3), and data not shown). Specificity of the staining was also confirmed by performing positive control IF microscopy analysis to detect expression of MHC-II, NOS2, and TNF-α or IL-6 after *Francisella novicida* infection. As expected, upon intranasal infection, bacteria-infected lung tissues displayed extensive staining for MHC-II, NOS2, and TNF-α (data not shown). Thus, the low to undetected levels of expression of M1-inflammatory molecules in microglia was specific to parasite infection and suggests a defect in the activation of these cells in helminth parasite-infected brains.

### HSF Inhibits Maturation and Activation of Microglia *In Vitro*

**Figure 2.**
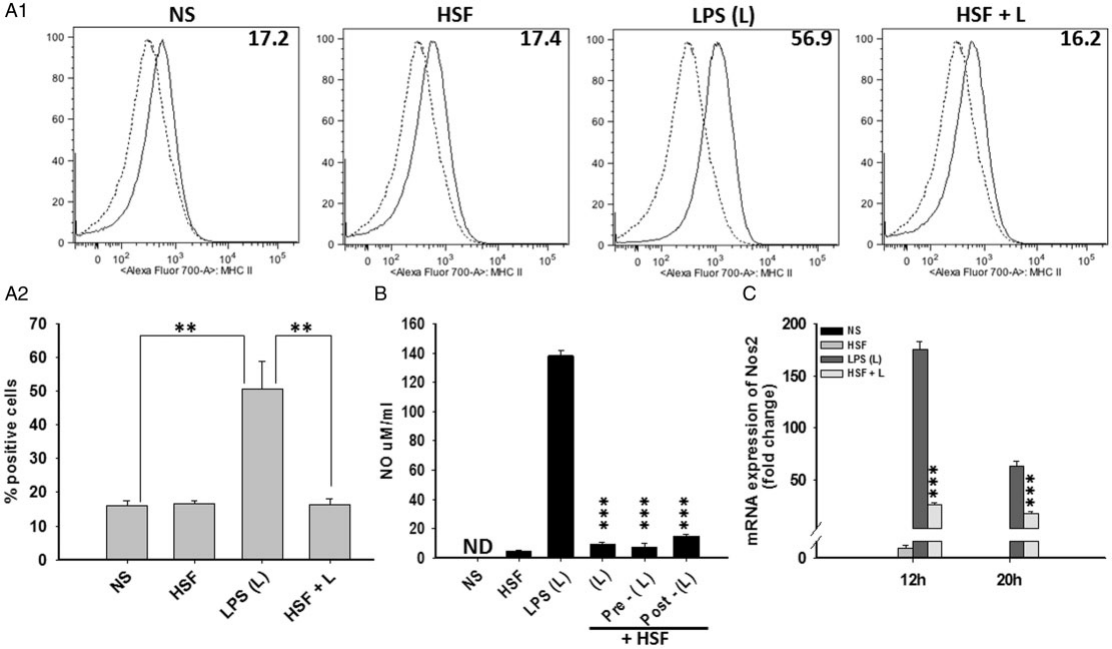
Helminth antigens downregulated agonist-induced maturation and activation of microglia. Primary microglia were pulsed with medium alone (NS), HSF at 25 µg/ml, LPS (L) at 10 ng/ml, or HSF at 25 µg/ml prior to the addition of LPS in the medium (HSF + L) and cultured for a total of 24-hr period. (A1) Microglia were harvested and flow cytometry analysis was performed to measure surface expression of MHC-II (A1). Dotted line in the histogram shows control staining with isotype/fluorochrome control Ab, and filled line shows signal with specific Ab (A1). (A2) Results obtained from flow cytometry analyses in (A1) are expressed as the mean ± *SEM* from three independent experiments. Significant differences in MHC-II-positive microglia after stimulations are denoted by asterisks (**p < .005). (B) Cells were pulsed with medium alone (NS), HSF at 25 µg/ml, LPS (L) at 10 ng/ml, or HSF added at 25 µg/ml to the culture together (HSF + L), prior (HSF + Pre-L) or after (HSF + Post-L) the addition of LPS in the medium (HSF + L) and cultured for a total of 24-hr period. NO contents in culture supernatants were detected by Griess reagent as recommended by the manufacturer (Promega). The mean ± *SE* concentration of NO in three independent experiments was determined. Significant differences were measured by Student’s *t* test (*p < *.001). ND- Not detected. (C) Microglia were pulsed with medium alone or HSF, LPS, HSF + LPS (L) for 12 hr, or 24 hr. RNA was isolated and reversed transcribed to cDNA by using random primers. Levels of NOS2 and housekeeping gene 18 S in these samples were measured by real-time PCR analysis as described using SYBR green as the detection dye. NOS2 mRNA level was normalized to the mRNA level of 18 S in the same sample, and fold change in mRNA expression of NOS2 after each treatment over their respective baseline control level in unstimulated samples was expressed in arbitrary units. Data shown are representative of three independent experiments. Significant differences were measured by Student’s *t* test (*p < *.001). HSF = helminth soluble/secreted factor; LPS = lipopolysaccharide; MHC = major histocompatibility complex; NO = nitric oxide; NOS2 = nitric oxide synthase 2; PCR = polymerase chain reaction.

Next, the effect of helminth molecules on maturation of primary microglia was analyzed *in vitro*. Flow cytometric analysis revealed that exposure to HSF itself did not affect the surface expression of the myeloid cell maturation marker MHC-II in microglia as compared with the untreated cells (Figure 2(A1) and (A2)). We have recently reported that helminth molecules inhibit LPS-induced maturation of bone marrow-derived macrophages (MФ; Chauhan et al., 2014). We thus examined the effect of HSF on LPS-induced MHC-II expression in primary microglia. Microglia stimulated with LPS alone exhibited an upregulated surface expression of MHC-II (Figure 2(A1) and (A2)). This LPS-induced activation of microglia was completely blocked upon exposure of cells to HSF (Figure 2(A1) and (A2)). Furthermore, these microglia treated with HSF alone, or when exposed to LPS in the presence and absence of HSF, displayed similar levels of viability as the medium alone (as examined by MTT assay), suggesting the specificity of the immune suppressive effect of HSF.

As NOS2 expression and subsequent NO production is a signature for M1-inflammatory phenotype of myeloid cells (Jenkins and Allen, 2010), we then tested the effect of HSF alone as well as on LPS-induced production of NO in microglia. LPS stimulation activated microglia to secrete large amounts (∼150 μM/ml and ∼250 μM at 24 and 48 hr post exposure) of NO (Figure 2(B), data not shown). In contrast, NO was undetected in culture supernatant of untreated or HSF-stimulated microglia (Figure 2(B)). Interestingly, coexposure to HSF completely inhibited the LPS-induced secretion of NO (Figure 2(B)). Interestingly, this blocking effect of HSF was independent of whether the cells were costimulated with HSF and LPS together or HSF was added before or after the addition of LPS to the culture (Figure 2(B)). Furthermore, HSF alone did not modulate gene expression of NOS2, the enzyme responsible for inducible NO production at both 12 hr and 20 hr post stimulation (Figure 2(C)). However, HSF also inhibited LPS-induced NOS2 mRNA expression (Figure 2(C)). Together, the results from these studies clearly demonstrate an inhibitory effect of HSF on microglia-associated M1-inflammatory response.

### HSF Downregulates Pol-II Recruitment at the Promoter of Inflammatory Mediators

**Figure 3.**
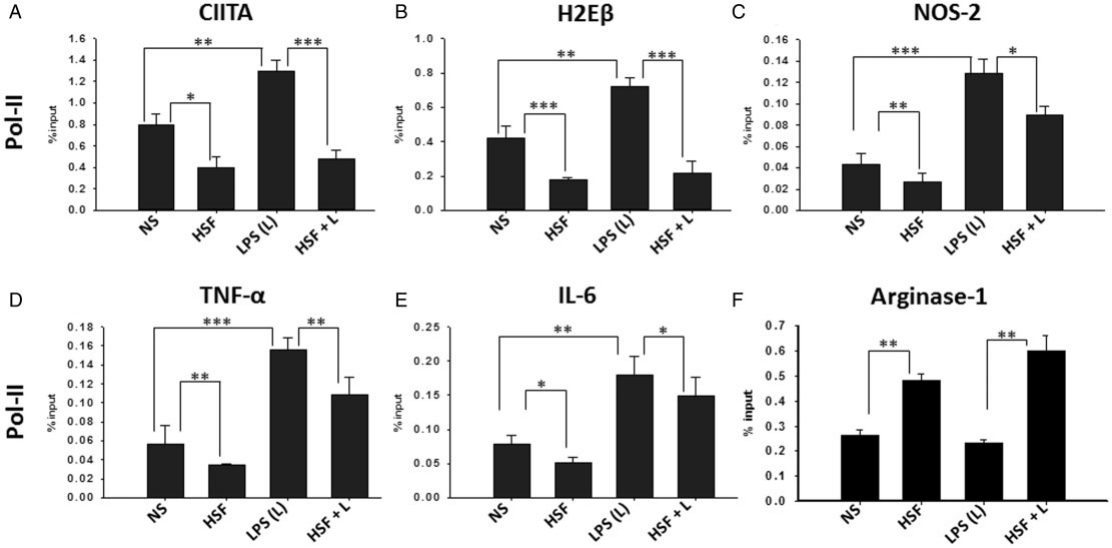
HSF-mediated modulation in Pol-II recruitment. Primary microglia were pulsed with medium alone (NS), HSF at 25 µg/ml, LPS at 10 ng/ml, or HSF at 25 µg/ml prior to the addition of LPS in the medium (HSF + L) and cultured for a total of 24-hr period. Cells were harvested, and ChIP was performed with anti-Pol-II or isotype IgG or with no antibody. Pol-II occupancy at the promoter regions of CIITA (A), MHC-II (H2Eβ) (B), NOS-2 (C), TNF-α (D), IL-6(E), and Arginase-1 (F) was assessed by qPCR and expressed as percentage of input. The mean ± *SE* of Pol-II occupancy using anti-Pol-II or isotype IgG (control) in three independent experiments was determined. Significant differences were measured by Student’s *t* test (*p < .05; **p < .005; *p < *.001). HSF = helminth soluble/secreted factor; Pol-II = polymerase II; LPS = lipopolysaccharide; ChIP = Chromatin Immunoprecipitation; MHC = major histocompatibility complex; NOS2 = nitric oxide synthase 2; TNF-α = tumor necrosis factor-alpha; IL-6 = interleukin-6; qPCR = quantitative polymerase chain reaction.

An important step in the regulation of gene expression is the recruitment of Pol-II along with the general transcription factors that enable the initiation of RNA synthesis (Rogatsky and Adelman, 2014). We next examined the effect of HSF on the recruitment of Pol-II to the promoter of MHC-II, CIITA, TNF-α, IL-6, and NOS2 using ChIP assays. As positive control, the level of Pol-II occupancy at the housekeeping GAPDH in the same sample was measured. As negative control, ChIP analysis using isotype IgG antibody was performed on the same samples. Compared with the untreated microglia, HSF-stimulated cells displayed a significantly decreased Pol-II occupancy at the promoter of all the immune mediators analyzed (MHC-II, CIITA, TNF-α, IL-6, and NOS2; Figure 3(A) to (E)). The effect of HSF on LPS-induced Pol-II recruitment to the promoter of the previously mentioned host mediators was examined. As expected, exposure to LPS significantly increased Pol-II occupancy at the promoter regions of MHC-II (Figure 3(B)), CIITA (Figure 3(A)), NOS2 (Figure 3(C)),TNF-α (Figure 3(D)), and IL-6 (Figure 3(E)), whereas minimal to no Pol-II occupancy was observed using the isotype IgG antibodies (data not shown). This effect of LPS was significantly inhibited in the presence of HSF (Figure 3). In contrast, HSF, LPS, HSF/LPS stimulation had no significant effect on Pol-II occupancy at the housekeeping gene GAPDH, as compared with the untreated microglia (data not shown). Moreover, as helminth infections are known to induce markers associated with M2 in helminth infections (Harn et al., 2009; Jenkins and Allen, 2010; Mishra et al., 2010), Pol-II occupancy was examined at the promoter regions of Arginase-1 (a signature marker of M2 activation phenotype). As compared with control cells, HSF-stimulated cells displayed a significantly increased Pol-II occupancy at the promoter of Arginase-1 (Figure 3(F)). In contrast, no difference in Pol-II occupancy was observed at the promoter of Arginase-1 after LPS exposure, which was significantly augmented in the presence of HSF (Figure 3(F)). Taken together, results from these experiments suggest that HSF inhibited Pol-II recruitment at the promoter of the inflammatory immune mediators in microglia.

### HSF Downregulates Positive Histone Marks at the Promoter of Inflammatory Mediators

**Figure 4.**
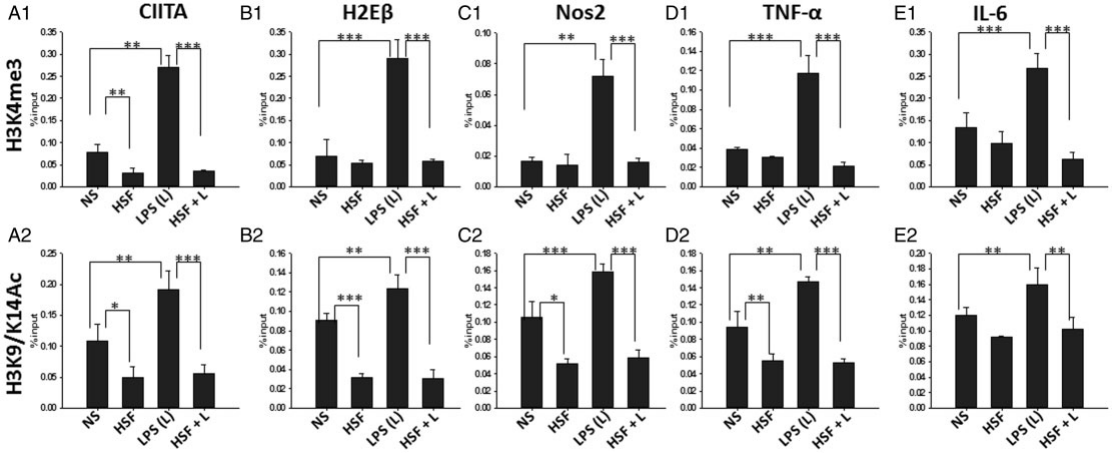
HSF downregulates positive histone marks. Primary microglia were pulsed with medium alone (NS), HSF at 25 µg/ml, LPS at 10 ng/ml, or HSF at 25 µg/ml prior to the addition of LPS in the medium (HSF + L) and cultured for a total of 24-hr period. Cells were harvested, and ChIP was performed with anti-H3K4Me3, or anti-H3K9/14Ac or isotype IgG or with no antibody. Presence of H3K4Me3 at the promoter regions of CIITA (A1), MHC-II (H2Eβ) (B1), NOS-2 (C1), TNF-α (D1), and IL-6(E1) was assessed by qPCR and expressed as percentage of input. Presence of H3K9/14Ac at the promoter regions of CIITA (A2), MHC-II (H2Eβ) (B2), NOS-2 (C2), TNF-α (D2), and IL-6 (E2) was assessed by qPCR and expressed as percentage of input. The mean ± *SE* of H3K4Me3 and H3K9/14Ac presence in three independent experiments was determined. Significant differences were measured by Student’s *t* test (*p < .05; **p < .005; *p < *.001). HSF = helminth soluble/secreted factor; LPS = lipopolysaccharide; ChIP = Chromatin Immunoprecipitation; MHC = major histocompatibility complex; NOS2 = nitric oxide synthase 2; TNF-α = tumor necrosis factor-alpha; IL-6 = interleukin-6; qPCR = quantitative polymerase chain reaction.

Chromatin states, defined by well-established combinations of histone marks, determine the magnitude of gene expression by cells in response to extracellular stimuli (Ernst et al., 2011; Zhou et al., 2011). Events involving histone marks lead to chromatin remodeling in the promotor-proximal, which in turn modulates Pol-II binding to the promoter. Positive histone marks such as H3K4me3 and H3K9/14Ac are essential for making the promoter region accessible to Pol-II and transcription factors (Natoli et al., 2011). Thus, the effect of HSF on the expression of H3K4me3 and H3K9/14Ac at the promoters of the previously mentioned inflammatory mediators was analyzed (Figure 4). ChIP analysis revealed that HSF alone downregulated H3K9/14Ac mark at the promoter region of all the inflammatory mediators analyzed (Figure 4(A1), (B1), (C1), (D1), and (E1)). Moreover, coculturing with HSF significantly inhibited the LPS-induced increase in the expression of H3K9/14Ac mark at the promoter region of MHC-II, CIITA, TNF-α, IL-6, and NOS2 (Figure 4(A1), (B1), (C1), (D1), and (E1)). In contrast, incubation of HSF alone had no significant measurable effect on H3K4Me3 at the promoters of most of the measured inflammatory mediators (Figure 4(B2), (C2), (D2), and (E2)) with the exception of CIITA (Figure 4(A2)) that significantly reduced by HSF alone as compared with the untreated microglia. However, as expected, exposure of LPS induced a significant increase in H3K4Me3 marks at the promoters of all of these M1-inflammatory mediators (Figure 4(A2), (B2), (C2), (D2), and (E2)). Costimulation of HSF and LPS substantially inhibited the LPS-induced increase in H3K4Me3 marks (Figure 4(A2), (B2), (C2), (D2), and (E2)). Further, ChIP analysis performed using isotype IgG antibody on the same samples display a very low reactivity (Figure 3, and data not shown). Together, these data suggest that chromatin modification by helminth antigens is an important immunosuppressive mechanism of inhibition of inflammatory gene expression.

## Discussion

NCC typically has a biphasic course: an initial phase of parasitic infection that is characterized with a lack of detectable CNS inflammation, followed by a symptomatic phase associated with hyper-inflammation (Mishra et al., 2009a; Alvarez et al., 2010b). Microglia often function as first responder cell type in the CNS that regulates the development of CNS inflammation (Nayak et al., 2012). Indeed, an imbalance of microglia activation resulting in deleterious and prolonged inflammation has been directly correlated with a majority of the neuropathological conditions (Ramesh et al., 2013a,^ 2013^b). In contrast, suppression of innate immune activation mechanisms of microglia may contribute to a downregulated inflammatory response that is characteristic of asymptomatic NCC. Thus, an improved understanding of microglia activation phenotypes and the mechanism underlying the downregulation of inflammatory gene expression in NCC is warranted. Here, we show that microglia in murine NCC fail to acquire a mature/activated phenotype, and the suppression of the signature inflammatory molecules by helminth parasite is regulated via alteration in Pol-II recruitment and positive histone marks at the promoter of these gene loci. Our results strongly suggest that helminth modulates epigenetic pathway to regulate microglial inflammation that may in turn be an important regulatory mechanism to block CNS inflammation in brain helminth infections, including NCC.

The present studies were designed to analyze the activation phenotype of microglia in murine NCC. We assessed the expression of M1 signature inflammatory cytokines TNF-α and IL-6 in microglia in the CNS during *M. corti* infection. In addition, we analyzed the expression of MHC-II, which is indicative of maturation of myeloid cells, and NOS2 that is a classical M1 myeloid activation phenotype marker (Kreider et al., 2007). Our results show that TNF-α and IL-6 were undetected in microglia. Moreover, expression of MHC-II and NOS2 was practically undetected in these cells in murine NCC brains. This is in line with our recent report where a similar trend of low/diminished expression of costimulatory molecules such as CD80 and CD86 was observed in infiltrating macrophages in brains of parasite-infected mice (Chauhan et al., 2014). We also reported that there is a low/undetectable level of MHC-II in many of the infiltrating macrophages in parasite-infected brains of NCC mice. The *in vitro* experiments using primary microglia similarly demonstrated inhibition of agonist-induced MHC-II and NOS2 expression by helminth factors. Together, these results clearly demonstrate that helminth parasite suppress M1-inflammatory activation in microglia. Microglia are critically positioned at the interface between innate immune activation-induced CNS inflammation and subsequent manifestation of adaptive immunity in the brain microenvironment. Thus, it is a reasonable hypothesis that dampening the TLR initiated inflammatory response is beneficial for parasitic growth and would also prevent collateral tissue damage due to excessive inflammation. Moreover, an immature microglial phenotype with low expression of MHC-II downregulates the proliferation of activated effector T cells and induces T cell anergy/regulatory phenotype (Latchman et al., 2001; Sharpe et al., 2007). Indeed, expansion of regulatory T cells in the CNS microenvironment has been reported in NCC patient (Adalid-Peralta et al., 2013). *In toto*, helminth-induced downregulation of innate immune pathway activation is likely crucial in establishment of chronic parasitic infections as well as for maintaining tissue homeostasis.

Gene transcription is a complex process, but the expression of gene loci in cells can be broadly divided into three distinct transitional steps (Rogatsky and Adelman, 2014). First, the promoter region surrounding the transcription start site must be accessible through the removal of repressive nucleosomes. Second, Pol-II and general transcription factors are recruited to facilitate the priming of RNA synthesis. Third, stimulation-specific transcriptional factors and chromatin regulators are recruited to the site as a result of activation, leading to Pol-II migration and productive transcription. Epigenetic events consisting of posttranslational modifications of DNA at the CpG motifs and DNA binding proteins such as histones, and noncoding RNA, have been demonstrated to regulate the previously mentioned three states of transcriptional activity thereby determining the kinetics and magnitude of gene expression (Zhou et al., 2011; Buecker and Wysocka, 2012). The majority of these studies has been focused on macrophages, and it is of note that epigenetic events modulating microglial gene expression in the context of CNS inflammation have been understudied. Among these events, histone modifications have received the most attention in efforts to understand the activation of inflammatory genes. Histone modifications are broadly divided into positive and negative marks that are typically correlated with the levels of productive and nonproductive gene expression, respectively (Ernst et al., 2011; Zhou et al., 2011). In the absence of inflammatory stimuli such as TLR ligation, inflammatory gene expression is restrained by the presence of gene-specific negative histone marks (Fang et al., 2012; Kruidenier et al., 2012; Zhu et al., 2012). Once the cell receives stimulation through TLR ligands, an increased expression of positive histone marks, such as H3K4Me3 and H3K9/14Ac, occurs at the promoter of the inflammatory cytokine gene loci (De Santa et al., 2009; Hargreaves et al., 2009; Escoubet-Lozach et al., 2011). These positive histone marks are thought to facilitate chromatin accessibility of genes and binding of factors such as Pol-II and signaling transcription factors, to promote transcription elongation (Adelman et al., 2009). Therefore, it is indeed interesting that we show here an inhibition of the LPS-induced upregulation of H3K4Me3 and H3K9/14Ac, as well as suppression of Pol-II recruitment at the promoter of inflammatory mediators after exposure to immune suppressive helminth-derived molecules. These findings are in line with earlier findings that demonstrated that intracellular protozoa parasite *Toxoplasma gondii* infection impairs LPS-induced chromatin remodeling at TNF-α promoter (Leng et al., 2009) in macrophages. However, unlike *Toxoplasma gondii* infection alone, in our studies helminth factors from *M. corti*, which is a macroscopic and extracellular cestode, by themselves downregulated H3K4Me3 and H3K9/14Ac expression, as well as reduced the Pol-II recruitment at the promoter of inflammatory mediators in microglia as compared with the unstimulated cells. This suggests that extracellular macroscopic helminth parasites, such as *M. corti* and *T. solium*, have likely acquired additional mechanisms to modulate epigenetic landscape to deactivate cells such as microglia. However, to conclusively ascertain this, it will be important to examine additional epigenetic mechanisms, including the distribution of other positive and negative histone marks, and their correlation with chromatin conformation and genome-wide gene expression profile in microglia.

In NCC, the loss of helminth-induced immune suppressive mechanisms upon parasite death is thought to result in the detrimental inflammatory response that contributes to the disease-associated neuropathology (White, 2000; Nash et al., 2006; Sciutto et al., 2007). Moreover, invasive macroscopic helminth parasites such as *M. corti* and *T. solium* often cause host tissue destruction because of their size as well as metabolic activity; however, an inflammatory response is undetected in the asymptomatic phase. TLRs act as sentinels not only for PAMPs, but also recognize damage-associated molecular patterns (DAMPs) from dead and damaged cells and tissues, resulting in inflammation (Sangiuliano et al., 2014). In this regard, our results presented here, along with previous reports (Chauhan et al., 2014), are of particular interest, as they demonstrate helminth factor-mediated downregulation of the TLR ligation-induced inflammatory response. We speculate that helminth-mediated regulation of epigenetic pathways is likely an important mechanism involved in the development of the asymptomatic phase. We also anticipate that specific helminth molecules differentially modulate the gene expression of inflammatory, anti-inflammatory, and tissue repairing factors by establishing distinct histone marks. Identifying which of the multitude of histone marks play specific functional role in determining genome-wide gene expression in microglia is a major focus of the current research in our laboratory.
